# Effects of major air pollutants on angina hospitalizations: a correlation study

**DOI:** 10.1186/s12889-024-19380-2

**Published:** 2024-07-15

**Authors:** Anning Zhu, Yongqin Cao, Chunlan Li, Jingze Yu, Miaoxin Liu, Ke Xu, Ye Ruan

**Affiliations:** 1 School of Public Health, Lanzhou University, Lanzhou, 730000 PR China; 2 Gansu Provincial Center for Disease Control and Prevention, Lanzhou, 730000 PR China; 3 Third People’s Hospital of Gansu Province, Lanzhou, 730000 PR China

**Keywords:** Angina pectoris, Air pollution, Hospitalization, Relative risk

## Abstract

**Background:**

Angina is a crucial risk signal for cardiovascular disease. However, few studies have evaluated the effects of ambient air pollution exposure on angina.

**Objective:**

We aimed to explore the short-term effects of air pollution on hospitalization for angina and its lag effects.

**Methods:**

We collected data on air pollutant concentrations and angina hospitalizations from 2013 to 2020. Distributed lag nonlinear model (DLNM) was used to evaluate the short-term effects of air pollutants on angina hospitalization under different lag structures. Stratified analysis by sex, age and season was obtained.

**Results:**

A total of 39,110 cases of angina hospitalization were included in the study. The results showed a significant positive correlation between PM_2.5_, SO_2_, NO_2_, and CO and angina hospitalization. Their maximum harmful effects were observed at lag0-7 (RR = 1.042; 95% CI: 1.017, 1.068), lag0-3 (RR = 1.067; 95% CI: 1.005, 1.133), lag0-6 (RR = 1.078; 95% CI: 1.041, 1.117), and lag0-6 (RR = 1.244; 95% CI: 1.109, 1.397), respectively. PM_10_ did not have an overall risk effect on angina hospitalization, but it did have a risk effect on women and the elderly. O_3_ was significantly negatively correlated with angina hospitalization, with the most pronounced effect observed at lag0-6 (RR = 0.960; 95% CI: 0.940, 0.982). Stratified analysis results showed that women and the elderly were more susceptible to pollutants, and the adverse effects of pollutants were stronger in the cold season.

**Conclusion:**

Short-term exposure to PM_2.5_, SO_2_, NO_2_, and CO increases the risk of hospitalization for angina.

**Supplementary Information:**

The online version contains supplementary material available at 10.1186/s12889-024-19380-2.

## Introduction

Angina pectoris (hereinafter referred to as angina) is primarily characterized by retrosternal chest pain, pressure, or discomfort, which is usually exacerbated by fatigue and emotional tension [1]. Symptoms of angina can greatly limit daily activities, negatively impacting quality of life and often leading to premature retirement in working-age patients [2]. Angina is primarily caused by an imbalance between myocardial oxygen supply and demand, with the most common cause being coronary artery disease, where atherosclerotic plaques narrow the vessel lumen supplying oxygen and nutrients to the myocardial cells [3]. Angina is a common manifestation and potential marker of coronary artery disease, which is a leading cause of death worldwide, with an annual mortality rate ranging from 1.6 to 3.2% [4–6]. More than half of patients with coronary artery disease present initially with angina, and the presence of chronic angina doubles the risk of major cardiovascular events [7, 8]. In the United States, approximately 10 million people suffer from angina, with more than 500,000 cases diagnosed each year [3]. In some developing countries, this health burden may be even more severe. Reducing the burden of angina is an important goal, and reducing the risk factors for angina and lowering the hospitalization rate for angina are top priority.

Ambient air pollution is recognized as a primary environmental risk factor for global health, especially in low- and middle-income countries [9]. It is well-known that hypertension, unhealthy diet, and fasting blood sugar are widely studied causes of angina [10]. In addition to these factors, changing lifestyle and reducing exposure to ambient air pollution alone can effectively treat angina [11, 12]. A systematic review indicated that short-term exposure to ambient air pollution is associated with an increased risk of angina incidence (including hospitalization and emergency department visits) [13]. Experimental studies have found that rabbits regularly exposed to PM exhibit more severe coronary artery disease with increased plaque size and more extensive aortic atherosclerosis [14]. Atherosclerosis is the main cause of angina. Therefore, it is necessary to investigate the impact of air pollution on angina in order to implement targeted prevention measures.

However, studies on the impact of air pollution on angina are relatively scarce, and are mainly focused on economically developed areas, with inconsistent results. The relationship between angina and air pollution may differ from that of other cardiovascular diseases. This study provides new scientific evidence on the impact of ambient air pollution on angina. Additionally, our research offers a scientific basis for the formulation of public health policies in Lanzhou city and serves as a reference for other similar regions. Lanzhou, located inland with a relatively underdeveloped economy, is one of the most seriously air-polluted cities in China. Its special valley terrain complicates the dispersion of air pollutants. The burden of angina in Lanzhou cannot be ignored, yet there have been no previous studies exploring the impact of air pollution on angina.

This study utilized distributed lag non-linear models (DLNM) to analyze the short-term effects and lag effects of air pollution exposure on angina hospitalizations among urban residents. Stratified analyses were conducted by sex, age, and season. Our aim is to provide scientific evidence for implementing measures to reduce the occurrence of angina.

## Materials and methods

### Study area

Lanzhou (longitude 102°36′∼104°35′ east and latitude 35°34′ ∼37°00′ north) is the provincial capital of Gansu Province, located in the northwest of China. As of 2023, Lanzhou city has a resident population of 4.425 million. It is an important industrial base and comprehensive transportation hub in the northwest region of China. The primary sources of air pollution in Lanzhou include industrial emissions and vehicle exhaust. Lanzhou is characterized as a typical river valley basin city with a high frequency of temperature inversions. The unique climatic features hinder the diffusion of pollutants, thus exacerbating air pollution.

### Hospitalizations data

The daily hospitalization data for angina from January 1, 2013, to December 31, 2020 were collected from all secondary and tertiary comprehensive hospitals in the main urban area of Lanzhou. China implements a three-level medical system, with secondary and tertiary hospitals are qualified to provide specialized care. The data include information such as sex, age, residential address, disease code, admission date, and discharge date. We screened the cases using the 10th revised version of the International Classification of Diseases (ICD-10) and included cases coded I20.0-I20.9 (angina pectoris) in this study. Logical corrections were applied to the hospitalization data, excluding cases with missing and duplicate information. Screening based on the registered residential addresses of patients was conducted to exclude patients residing outside Lanzhou, ensuring that the study population comprised permanent residents of Lanzhou.

### Air pollution and meteorological data

During the study period, air pollution data were collected from three national air quality monitoring stations within the study area, namely the Gansu Provincial Construction Staff Hospital station, the Railway Design Institute station, and the Biological Products Research Institute station. Hourly measurements reported by these three monitoring stations were averaged to calculate daily mean concentrations of PM_2.5_, PM_10_, SO_2_, NO_2_, O_3_, and CO. O_3_ concentration are 8-hour daily maximum averages, while concentrations of other air pollutants are 24-hour averages. The national air quality monitoring stations are set up in accordance with the National Environmental Protection Standards of the People’s Republic of China (HJ 664–2013) and are located away from major roads, industrial sources and pollutants. Pollutant concentrations were measured in accordance with the Ambient Air Quality Standards (GB 3095 − 2012) issued by the State Environmental Protection Administration (SEPA), and the results of the measurements can reflect the overall air pollution level in our study area. Therefore, the average of the data from the three air quality monitoring stations provides a good representation of the exposure levels of the population.

We collected daily meteorological data, including daily mean temperature (°C) and average relative humidity (%) during the study period from the China Meteorological Data Service Center (http://data.cma.cn). These two variables were introduced into the model to control for confounding factors.

### Statistical analysis

Descriptive statistical analyses were conducted on the concentration of air pollutants, meteorological factors, and hospitalization for angina during the study period. Data were described as mean, standard deviation (SD), minimum (Min), maximum (Max), median and quartile. Spearman correlation analysis was performed to assess the correlation between air pollutants and meteorological factors. Considering that the impact of air pollution on disease is a non-linear effect with a certain degree of persistence and lag. In addition, the occurrence of daily angina hospitalizations is a small probability event relative to the total population with a distribution that approximates a Poisson distribution. Therefore, we employed a combination of Distributed Lag Non-linear Models (DLNM) and Generalized Additive Models (GAM) to investigate the nonlinear and lagged effects of air pollution on angina. The model was constructed as:

$$\eqalign{Log(\mu{t})&=\alpha+\beta{X}_{t,l}\,+ns(Temp_{t,}\,df=3)+ns(RH_{t,}\,df=3)\cr&\quad+ns(Time_{,}\,df=7)+DOW+Holiday}$$

where *t* is the observation day; *µt* refers to the number of hospitalizations for angina on day *t*; *Log(µt)* represents the expected value of the number of angina hospitalizations on day *t*; *α* represents the intercept; *l* is the maximum lag days; *X*_*t, l*_ is the cross-base matrix of air pollutants; *β* represents the matrix coefficient; *ns* represents the natural smoothing spline function; *Temp*_*t*_ is the average temperature on day *t*; *RH*_*t*_ is the relative humidity on day *t*; *Time* is the time variable; *DOW* is a dummy variable representing a control for weekly effects; *Holiday* is a dummy variable representing a control for public holidays. This study adopted the Quasi-Akaike Information Criterion (Q-AIC) to evaluate model fitting and synthesize prior literature to determine the best model with the most appropriate degrees of freedom. A natural spline function with degrees of freedom set to 3 is used to control temperature and relative humidity. The degree of freedom of the time function is set to 7 to control long-term trends and seasonality. In our study, we choose degrees of freedom based on the minimum value of Q-AIC, and the smaller the Q-AIC value, the better the model.

We used the single-pollution model to examine the short-term effects of air pollutants on angina hospitalization. We set the maximum lag time to 7 days and observe the effects of different lag days, including single-day lag (from lag0 to lag7) and cumulative lag (from lag0-1 to lag0-7). We plotted contour figures of relative risk values for angina hospitalizations against changes in air pollutant concentrations across different lag days to better understand the exposure-response-lag relationship. Subgroup analyses were further conducted to evaluate the effects of air pollutants on different ages (< 65 years old; ≥65 years old) [15, 16], sexes (man; woman), and seasons (cold season from October to March of the following year; warm season from April to September) [17]. Effect estimates are expressed as the relative risk (RR) of angina hospitalization per 10 µg/m^3^ increase in PM_2.5_, PM_10_, SO_2_, NO_2_, O_3_, and per 1 mg/m^3^ increase in CO, and the corresponding 95% confidence intervals (95% CI). The 95% CI does not include 1 indicates that the result is statistically significant.

In order to test the reliability and stability of the results, two methods of sensitivity analysis were used in this study. (1) The lag day with the maximum estimated impact for each pollutant in the single-pollutant model were selected, fitted the two-pollutant model, and compared the results of the single-pollutant and two-pollutant models to validate the reliability and stability of the model. (2) Change the degree of freedom (*df*) of the time variable (*df* = 6–10) and observe the change in RR value. If there is no notable change, it indicates that the model is robust, and the results are reliable. The R software version 4.2.2 was used for all statistical analyses, while the “dlnm” package was used to fit the models. Two-sided test with *P* values less than 0.05 were considered statistically significant.

## Results

### Description of the general situation

Descriptive statistics on air pollutant concentrations, meteorological factors, and daily angina hospitalizations are summarized in Table 1. Between January 1, 2013, and December 31, 2020, a total of 39,110 angina hospitalizations were collected in this study, with an average of approximately 13 hospitalizations per day. There were 24,119 (61.67%) man cases and 14,991 (38.33%) woman cases. In addition, 19,268 cases (49.27%) were ≥ 65 years old and 19,842 (50.73%) were < 65 years old. The mean daily number of admissions was higher in the cold season 13.66 (SD: 15.35) than in the warm season 13.11 (SD: 13.70). The average concentrations of PM_2.5_, PM_10_, SO_2_, NO_2_, O_3_, and CO during the study period were 49.75 µg/m^3^, 114.66 µg/m^3^, 21.65 µg/m^3^, 45.89 µg/m^3^, 83.77 µg/m^3^, and 1.16 mg/m^3^, respectively. The average daily temperature and relative humidity during the study period were 11.14℃ and 51.19%.

**Table 1:** Descriptive statistics of air pollutants, meteorological factors and hospitalizations for angina in Lanzhou, China, 2013–2020

Variable	Mean	SD	Min	*P*_25_	Median	*P*_75_	Max
**Air pollutants**							
PM_2.5_ (µg/m^3^)	49.75	29.27	0.00	31.00	42.78	60.83	278.00
PM_10_ (µg/m^3^)	114.66	83.12	0.00	70.00	98.84	136.23	1484.54
SO_2_ (µg/m^3^)	21.65	14.47	0.00	10.86	17.60	28.61	115.51
NO_2_ (µg/m^3^)	45.89	17.95	0.00	34.00	44.58	54.63	146.60
O_3_ (µg/m^3^)	83.77	43.57	8.00	53.00	76.00	107.00	462.00
CO (mg/m^3^)	1.16	0.69	0.00	0.70	0.96	1.41	4.65
**Meteorological factors**							
Mean temperature (℃)	11.14	9.88	-12.30	2.25	12.60	19.79	30.40
Relative humidity (%)	51.19	15.29	11.71	40.00	51.86	62.00	96.09
**Sex**							
Man	8.25	9.12	0.00	2.00	5.00	12.00	66.00
Woman	5.13	5.93	0.00	1.00	3.00	7.00	48.00
**Age**							
< 65 years	6.59	7.46	0.00	1.00	4.00	9.00	55.00
≥ 65 years	6.79	7.63	0.00	2.00	4.00	10.00	60.00
**Total hospitalization**	13.38	14.55	0.00	3.00	8.00	19.00	109.00
**Season**							
Warm	13.11	13.70	0.00	3.00	8.00	18.00	109.00
Cold	13.66	15.35	0.00	3.00	8.00	20.00	90.00

SD: standard deviation; *P*_*25*_: 25% quartile; *P*_*75*_: 75% quartile; Min: minimum; Max: maximum

Table S1 shows the spearman correlation coefficients between air pollutants, temperature, and relative humidity. The range of spearman correlation coefficients of PM_2.5_, PM_10_, SO_2_, NO_2_, and CO are significantly positively correlated with each other. O_3_ was negatively correlated with PM_2.5_, PM_10_, SO_2_, and CO, and had no correlation with NO_2_. Temperature was significantly positively correlated with O_3_ and significantly negatively correlated with other pollutants. Relative humidity had no correlation with CO and was significantly negatively correlated with other pollutants. The strongest correlation was between PM_2.5_ and PM_10_ (*r* = 0.850).

**Fig. 1:**
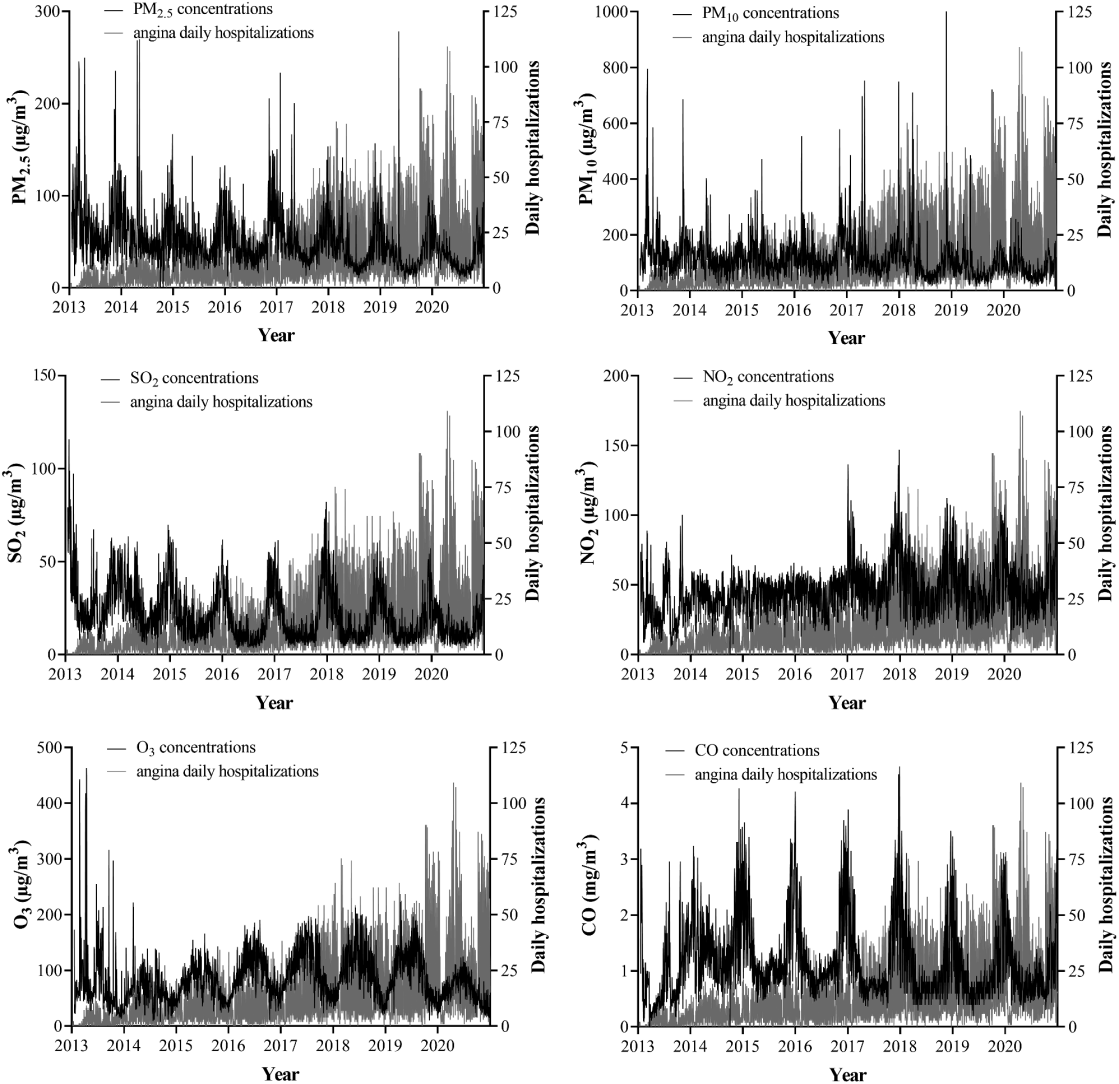
Time series of angina hospitalizations and pollutant concentrations in Lanzhou, China, 2013–2020

Figure 1 shows a time series plot of daily angina hospitalizations and air pollutant concentrations during the study period. Hospitalizations for angina increase yearly and fluctuate seasonally. Slightly more patients are hospitalized for angina in the cold season than in the warm season. The daily average concentrations of PM_2.5_, PM_10_, SO_2_ and CO show a decreasing trend year by year, while NO_2_ and O_3_ show an increasing trend year by year. PM_2.5_, PM_10_, SO_2_, NO_2_, and CO had higher concentrations in the cold season and lower concentrations in the warm season, while O_3_ showed the opposite trend. Trends in angina hospitalizations and air pollutant concentrations were generally consistent, especially during the cold season.

### Short-term effects of air pollutants on angina

**Fig. 2:**
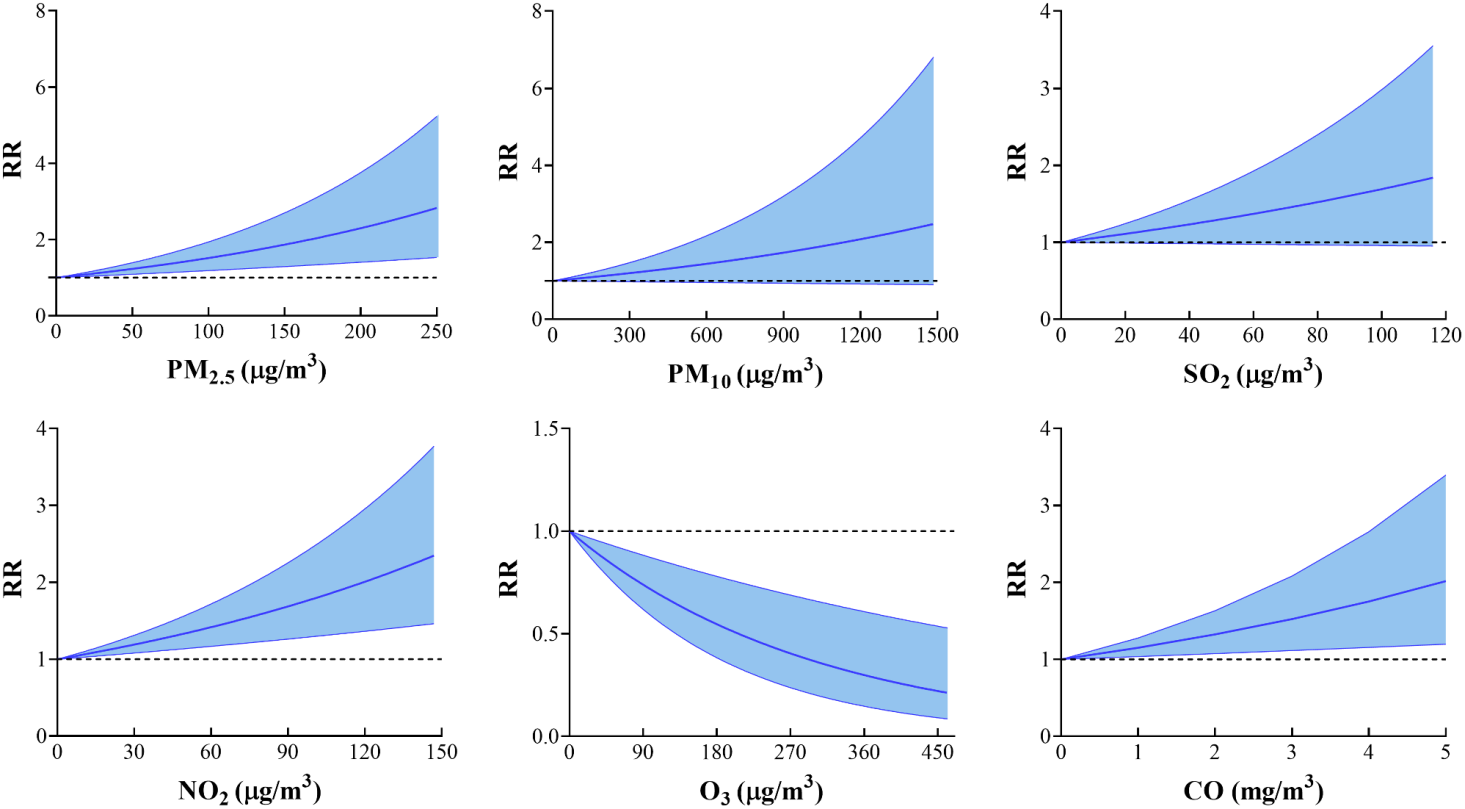
Exposure response relationships between six air pollutants and angina hospitalizations

Figure 2 shows the exposure-response relationship curves for air pollutants and hospitalization for angina. The relationship between them shows a linear curve without a threshold. The relative risk of angina hospitalization tended to increase with increasing concentrations of PM_2.5_, PM_10_, SO_2_, NO_2_, and CO. However, the relative risk of angina hospitalization tended to decrease with increasing O_3_ concentrations.

**Fig. 3:**
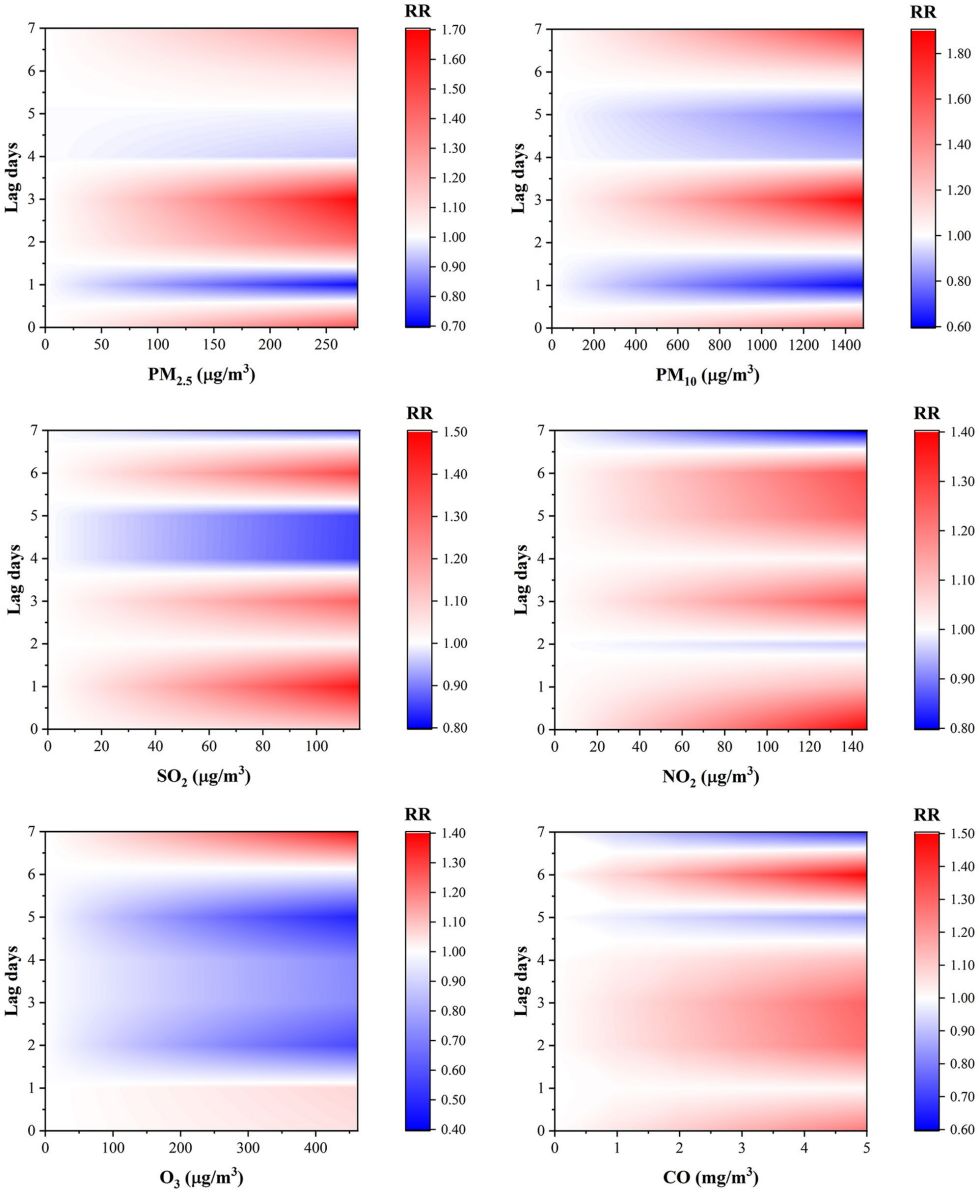
Contour plot of relative risk of angina hospitalization with changes in air pollutant concentration and lag days

A contour plot of the relative risk of angina hospitalization for lag0 to lag7 days of exposure to air pollution is shown in Fig. 3. The concentration lag effect showed that the association between pollutant concentration and angina hospitalization has non-linear and lagged effects. The risk of angina hospitalization increases as the concentration of pollutants increases. However, the trends varied among pollutants and showed different effects at different lag days.

**Fig. 4:**
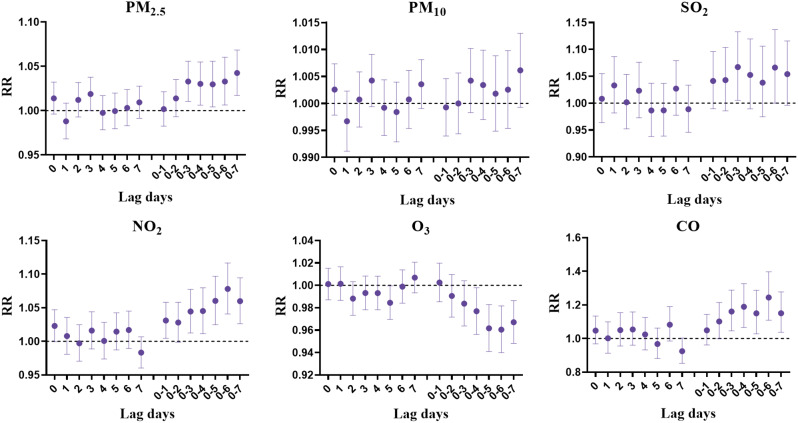
Relative risk (95% CI) of angina hospitalization for each 10 µg/m^3^ increase in air pollutant concentration (each 1 mg/m^3^ increase in CO) in single-day lags and cumulative lags

Figure 4 and Table S2 summarizes the relative risks (RR) and 95% confidence intervals (95% CI) for angina hospitalizations at different lag days in the single-pollutant model. PM_2.5_, SO_2_, NO_2_ and CO were significantly positively associated with angina hospitalization. PM_2.5_ had significant effects at lag3, lag0-3 to lag0-7 days, with the largest effect at lag0-7 (RR = 1.042; 95% CI: 1.017, 1.068). SO_2_ had significant effects at lag0-3 and lag0-6 days, with the largest effect at lag0-3 (RR = 1.067; 95% CI: 1.005, 1.133). NO_2_ had significant effects at lag0, lag0-1 and lag0-3 to lag0-7 days, with the largest effect at lag0-6 (RR = 1.078; 95% CI: 1.041, 1.117). CO had significant effects at lag0-3 to 0–7 days, with the largest effect at lag0-6 (RR = 1.244; 95% CI: 1.109, 1.397). In contrast, O_3_ was significantly negatively associated with angina hospitalization at lag5, lag0-4 to lag0-7 days, with the largest effect at lag0-6 (RR = 0.960; 95% CI: 0.940, 0.982). However, no correlation between PM_10_ and angina hospitalization was found.

### Subgroup analysis

**Fig. 5:**
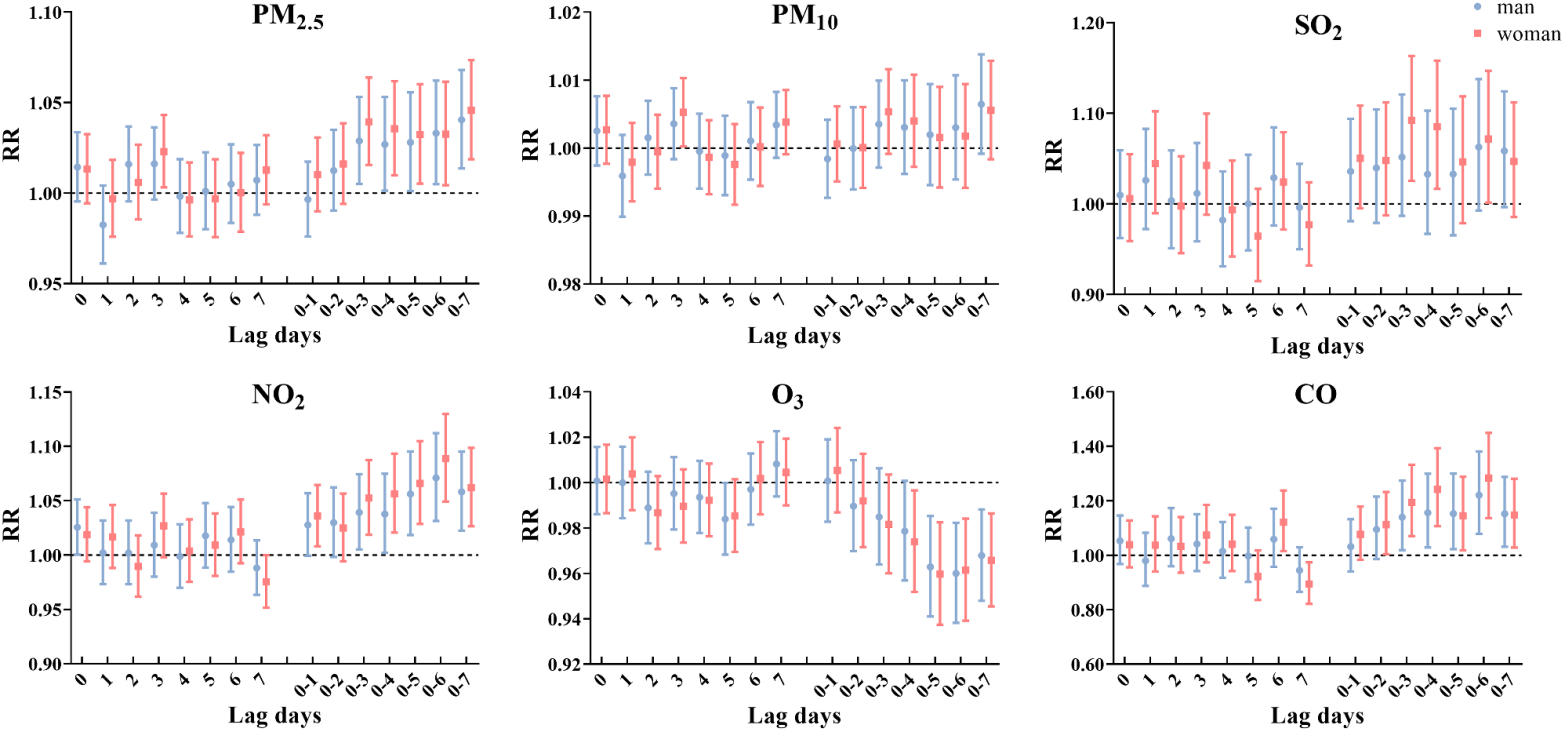
Relative risk (95% CI) of angina hospitalization stratified by sex for each 10 µg/m^3^ increase in air pollutant concentration (each 1 mg/m^3^ increase in CO) in single-day lags and cumulative lags

Figure 5 and Table S3 shows the RR (95% CI) of angina hospitalization stratified by sex at different lag periods. Among the six pollutants, PM_2.5_, NO_2_, and CO had a greater effect on angina in women than in men. The effects of PM_2.5_ at lag0-7 (RR = 1.046; 95% CI: 1.019, 1.073), NO_2_ at lag0-6 (RR = 1.089; 95% CI: 1.049, 1.130), and CO at lag0-6 (RR = 1.284; 95% CI: 1.136, 1.450) on angina hospitalization in women were the maximum. PM_10_ and SO_2_ only had effects on angina hospitalization in women. PM_10_ at lag3 (RR = 1.005; 95% CI: 1.000, 1.010) and SO_2_ at lag0-3 (RR = 1.092; 95% CI: 1.026, 1.163) had the greatest effect on angina hospitalization in women. No positive correlation was observed between O_3_ and angina hospitalization in both men and women.

**Fig. 6:**
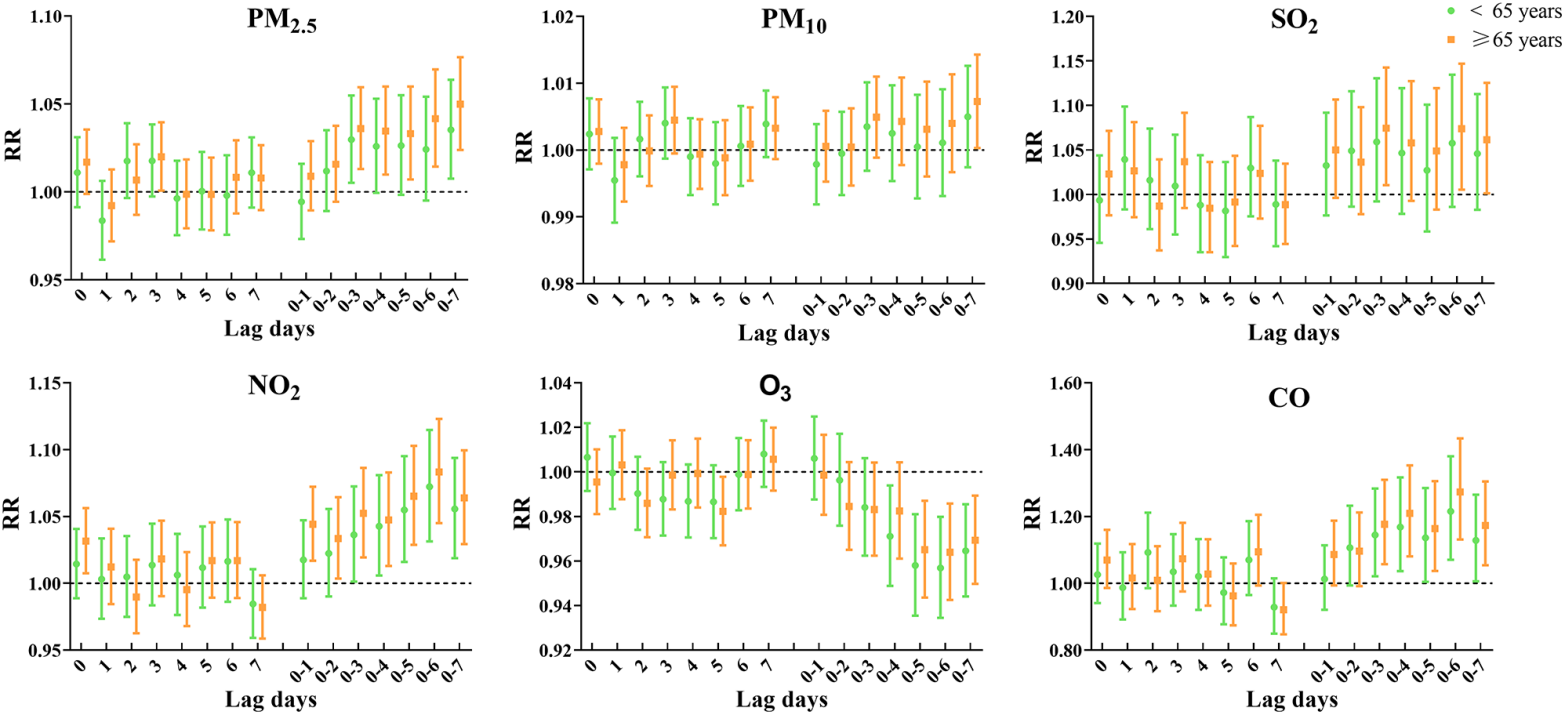
Relative risk (95% CI) of angina hospitalization stratified by age for each 10 µg/m^3^ increase in air pollutant concentration (each 1 mg/m^3^ increase in CO) in single-day lags and cumulative lags

Figure 6 and Table S4 shows the effect of air pollutants at different lags on angina hospitalization in different age groups. Individuals aged ≥ 65 years were more susceptible to PM_2.5_, PM_10_, SO_2_, NO_2_, and CO than those aged < 65 years. No effect of PM_10_ and SO_2_ on angina hospitalization at < 65 years was found. In the age ≥ 65 years group, PM_2.5_ at lag0-7 (RR = 1.050; 95% CI: 1.024, 1.077), PM_10_ at lag0-7 (RR = 1.007; 95% CI: 1.000, 1.014), SO_2_ at lag0-6 (RR = 1.074; 95% CI: 1.005, 1.147), NO_2_ at lag0-6 (RR = 1.083; 95% CI: 1.045, 1.123) and CO at lag0-6 (RR = 1.273; 95% CI: 1.131, 1.433) had the greatest effects on angina hospitalization. No significant risk of O_3_ for angina hospitalization was observed in either age group.

**Fig. 7:**
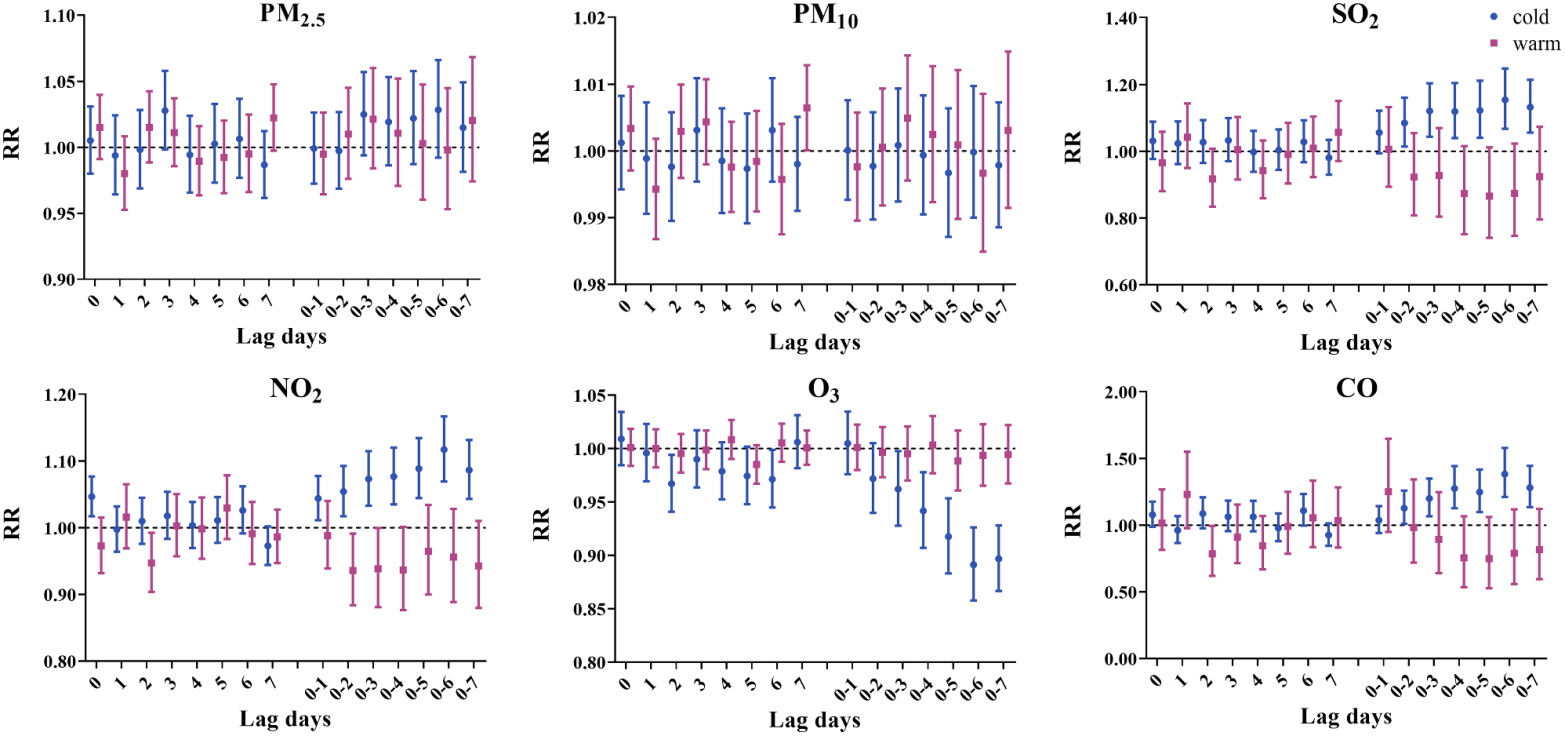
Relative risk (95% CI) of angina hospitalization stratified by season for each 10 µg/m^3^ increase in air pollutant concentration (each 1 mg/m^3^ increase in CO) in single-day lags and cumulative lags

Figure 7 and Table S5 shows the effect of changes in the concentration of six pollutants on hospitalization for angina in different seasons. SO_2_, NO_2_, and CO were significantly positively associated with angina hospitalization only during the cold season, with the largest associations occurring at lag0-6 (RR = 1.155; 95% CI: 1.068, 1.249), lag0-6 (RR = 1.117; 95% CI: 1.069, 1.167), and lag0-6 (RR = 1.384; 95% CI: 1.212, 1.580), respectively. PM_10_ was only associated with angina hospitalization during the warm season, with the maximum RR occurring at lag7 (RR = 1.006; 95% CI: 1.000, 1.013). O_3_ was significantly negatively associated with angina hospitalization only during the cold season, with the largest effect in lag0-6 (RR = 0.891; 95% CI: 0.858, 0.926).

### Sensitivity analysis

We selected the lag days with the maximum effect of each pollutant in the single-pollutant model and constructed two-pollutant models to verify the stability of the model (Table S6). Only the two-pollutant model constructed with SO_2_ had a lower effect on angina hospitalization than the single-pollutant model. The associations of the other pollutants with angina hospitalizations remained stable and like the results of the single-pollutant model. PM_2.5_ and PM_10_ did not coexist in the model due to their high correlation.

We assessed the robustness of the model by changing the degrees of freedom of the time variable from 6 to 10 (Table S7). The results indicate minimal variation in the RR values of each pollutant within the range of *df* from 6 to 10, suggesting robustness in model selection. Despite the fluctuation in *df* for time variable, the consistency in model outputs ensures the reliability of the results.

## Discussion

This study used time series analysis to investigate the short-term effects of exposure to air pollution on angina hospitalization and its lagged effects from 2013 to 2020 in Lanzhou. The results showed that short-term exposure to PM_2.5_, SO_2_, NO_2_, and CO increased the risk of angina hospitalization after controlling for confounders, and there was a significant lagged effect. Additionally, the impact of air pollution on angina hospitalization varied by sex, age, and season. Women and the elderly were more susceptible to pollutants, and the adverse effects of pollutants were stronger in the cold season. In summary, our findings further support that air pollution increases the likelihood of angina hospitalization.

During the study period, improvements in governance led to an overall decrease in the concentration of ambient air pollutants in Lanzhou. However, the concentrations of O_3_ and NO_2_ increased, due to the continuous growth in the number of automobiles in recent years, resulting in significant vehicle emissions. The concentrations of PM_2.5_, PM_10_, SO_2_, NO_2_, and CO were higher in winter and lower in summer, primarily due to the increased air pollution from fuel combustion for heating during the winter. Additionally, winter conditions are often dominated by cold high-pressure systems, which limit air movement and hinder pollutant dispersion. As a result, industrial emissions and residential heating emissions tend to accumulate over the city, further exacerbating air pollution. In contrast, O_3_ exhibited an opposite seasonal pattern, with higher concentrations in summer and lower concentrations in winter. This is likely because ozone is produced through several atmospheric reactions involving volatile organic compounds (VOCs), nitrogen oxides (NOx), and sunlight. These indirect ozone production processes are more efficient in warm weather [18].

In this study, we found that short-term exposure to PM_2.5_ increases the risk of angina hospitalization, with the greatest effect observed at lag0-7 (RR = 1.042; 95% CI: 1.017, 1.068). Similarly, many studies have identified an association between PM_2.5_ and angina. A study conducted in Beijing indicated that for hospitalizations due to angina, the odds ratio (OR) for PM_2.5_ severe pollution events lasting three days or more was 1.112 (95% CI: 1.095–1.130) [19]. Another study conducted in Canada showed that for every 10 µg/m³ increase in PM_2.5_, the prevalence rate (PR) of angina increased by 1.20 (95% CI: 1.09–1.31), and the incidence rate (IR) increased by 1.10 (95% CI: 0.97–1.24) [20]. Additionally, a study conducted in Poland found that PM_2.5_ had a harmful effect on angina at lag1 day, with a RR of 1.0066 (95% CI: 1.0058–1.0073) [21]. Contrary to our findings, some studies have not found an association between PM_2.5_ and hospitalization or emergency visits for angina [22, 23]. The small size and large specific surface area per unit of PM_2.5_ allow these particles to enter the systemic and pulmonary circulation [24, 25]. Additionally, heavy metals can bind to PM_2.5_ and adversely affect human health [26]. It is hypothesized that PM_2.5_ exerts its effects through three mechanisms: increased oxidative stress [27], activation of the immune system’s inflammatory response [28], and autonomic nervous system imbalance [29]. These mechanisms ultimately promote endothelial dysfunction, atherosclerosis, and systemic inflammation, leading to coronary artery spasm and reduced coronary blood flow, which can trigger angina attacks [3].

Our study found no correlation between PM_10_ exposure and angina hospitalization. This is consistent with the findings of a cohort study conducted in Europe, which showed that for every 10 µg/m^3^ increase in PM_10_, the relative risk of angina hospitalization at lag0 was 1.008 (95% CI: 0.986, 1.032), indicating no significant correlation [30]. Additionally, a time-series study conducted in Iran found no significant association between PM_10_ and hospitalization for angina, with a relative risk of 1.00451 (95% CI: 0.99998, 1.00906) at lag1 day [31]. Similarly, a study conducted in Canada also reported no correlation between emergency visits for angina and PM_10_ [22]. Larger particles such as PM_10_ typically cannot penetrate the airways and lungs due to their size, resulting in less direct health impacts [32]. However, studies have also highlighted the adverse health effects of PM_10_. A study on the Polish Smog indicated a significant association between PM_10_ concentration and cardiovascular disease mortality [33]. During Saharan dust events, each 10 µg/m³ increase in PM_10_ was associated with an 8.4% increase in daily mortality (95% CI: 1.5-15.8%). In contrast, there was no increase in daily mortality associated with PM_2.5_ [34]. The inconsistency in short-term risk associated with PM_10_ exposure may stem from variations in emission sources, chemical compositions, weather conditions, and population characteristics. In the future, there is a need for more precise experimental studies to explore the mechanisms underlying the impact of environmental particulate matter exposure on angina.

This study found a significant positive correlation between hospitalization for angina and SO_2_, with the largest effect at lag0-3 (RR = 1.067; 95% CI: 1.005, 1.133). There is now compelling evidence suggesting that exposure to SO_2_ exacerbates angina. A study conducted in Canada found that for every 5.1 ppb increase in SO_2_ at lag1 day, there was a 2.1% increase in angina emergency visits (95% CI: 0.2, 4.0) [22]. However, some studies have reported contradictory results. For instance, studies conducted in Iran and Shanghai found no significant association between angina hospitalization and SO_2_ [31, 35]. Fossil fuel combustion is the primary source of SO_2_, especially fuels used in diesel engines and power plants. The significant burning of high-sulfur coal led to the infamous 1952 London smog, predominantly composed of SO_2_ [36]. During and after the smog event, there was a high correlation between the levels of SO_2_ and the use of emergency beds by patients with heart and respiratory diseases [37]. Exposure to SO_2_ leads to an increase in Ang II in myocardial tissue, stimulating endothelin-induced coronary artery constriction, reducing capillary density, and increasing myocardial ischemia and hypoxia damage, thereby triggering angina [38]. Additionally, inhaled SO_2_ undergoes hydration in the respiratory tract, forming sulfurous acid, which subsequently decomposes into sulfites and bisulfites. These derivatives can be absorbed into the bloodstream, exerting toxic effects on the cardiovascular system [38].

In this study, NO_2_ was significantly positively associated with angina hospitalization, with the greatest impact observed at lag0-6 (RR = 1.078; 95% CI: 1.041, 1.117). Similarly, a study conducted in Europe found a positive association between an 8 µg/m^3^ increase in NO_2_ and same-day angina hospitalizations (RR = 1.032; 95% CI: 1.006, 1.058) [30]. A study in Poland reported that NO_2_ exposure increases the risk of hospitalization for unstable angina [21]. Studies conducted in Canada and Shanghai also found a significant positive correlation between NO_2_ and angina emergency visits [22, 35]. Although our findings align with these studies, the estimated effects differ, likely due to variations in pollutant units and study designs. NO_2_ is emitted from vehicle exhaust and stationary sources such as power plants and industrial boilers. Toxicological evidence indicates that inhaling NO_2_ can induce the overexpression of pro-inflammatory cytokines such as ICAM-A, COX-2, IL-1β, TNF-α, and iNOS [39]. Repeated exposure to NO_2_ can impair acetylcholine-mediated coronary artery dilation [40]. These mechanisms are closely related to the occurrence of angina.

We observed a significant association between CO exposure and hospital admissions for angina, with the highest relative risk observed at lag0-6 (RR = 1.244; 95% CI: 1.109, 1.397). CO is a well-recognized cardiovascular toxin, and its relationship with angina has been widely studied. A study conducted in Iran reported that for every unit increase in CO levels, hospital admissions increased by 1.00934 (95% CI: 1.00359–1.01512) [31]. Another study in São Paulo found that among air pollutants, only CO was associated with visits for angina [41]. In a multi-city survey in Canada, CO was not associated with emergency visits for angina in any of the seven cities individually, but in the pooled analysis, CO was positively associated with angina at lag0 day [22]. CO is a colorless, odorless gas produced by the incomplete combustion of fossil fuels. Hemoglobin’s affinity for CO is more than 200 times greater than its affinity for oxygen, so exposure to CO results in CO binding with hemoglobin, thereby reducing oxygen transport and causing varying degrees of hypoxia [42]. In addition to hypoxia, impaired myoglobin function, reactive oxygen species production, and interruption of the terminal oxidase in the electron transport chain can also lead to cardiac dysfunction [43, 44]. The precise biological mechanisms underlying the harmful effects of CO on angina require further investigation.

Interestingly, our study found a significant negative correlation between O_3_ exposure and angina hospitalizations. Some studies coincide with our findings. A time-series study conducted in Canada found that each 18.4 ppb increase in O_3_ was associated with a 3.0% (95% CI: 0.0, 5.9) decrease in angina emergency visits at lag1 day [22]. Similarly, a time-series study in Iran reported a negative association between O_3_ and angina hospitalizations, with an RR of 0.96396 (95% CI: 0.94256, 0.98582) [31]. O_3_, as a secondary pollutant, is produced through photochemical reactions between NOx and VOCs from combustion by-products [45]. Some research suggests that O_3_ can combine with O_2_ and locally increase eNOS activity, thereby protecting the heart from myocardial infarction. Furthermore, O_2_-O_3_ therapy has been shown to promote faster recovery and enhance healing processes, potentially preventing complications such as cardiovascular events in peripheral arterial disease [46]. Additionally, ozonated autohemotherapy has been used in the treatment of ischemic diseases, such as acute cerebral infarction [47, 48]. However, the effects of O_3_ on cardiovascular health appear to be unclear and even contradictory. Epidemiological studies provide evidence that short-term exposure to ozone increases the risk of angina hospitalizations [49, 50]. In Los Angeles, O_3_, a component of photochemical smog, is associated with deaths from respiratory and heart diseases [51]. Paradoxically, another study in Los Angeles found that O_3_ was either negatively correlated or not significantly associated with cardiovascular hospitalization rates [52]. In the lungs and circulatory system, O_3_ induces inflammation by releasing IL-2 and TNF-α, triggers oxidative stress through the direct oxidation of biomolecules and secondary formation of reactive oxygen species, and increases the level of the vasoconstrictor ET-1 [53]. The impact of air pollution on acute cardiovascular events is multifaceted, interactive, and complex, thus it is premature to conclude that O_3_ has a protective effect against ischemic stroke. Further research is needed to explore the underlying mechanisms.

The stratified analysis in this study indicates that women are more sensitive to air pollution than men, consistent with previous research findings [54, 55]. Several possible explanations have been proposed for this phenomenon. Earlier studies suggest that differences in body structure may contribute to sex disparities. Compared to men, women typically have smaller airways and more reactive airway responses. Additionally, women tend to have smaller coronary arteries and are more prone to diffuse atherosclerosis and microvascular dysfunction than men [56]. Another plausible reason could be the different social statuses and personal characteristics (such as daily life and working conditions) that make women more susceptible to the effects of air pollution. Moreover, studies have found a significant sex difference in the prevalence of angina, with women having a higher risk (6.7%) compared to men (5.7%) [57]. These factors may explain why women are more adversely affected by air pollution, leading to higher incidences of angina. In contrast, one study identified men as a more vulnerable group [58]. The differences in angina events between men and women during exposure to air pollution warrant further investigation.

We also observed differences between age groups. In this study, individuals over 65 were more susceptible to the adverse effects of air pollution, aligning with findings from some studies [35, 59]. As people age, they undergo physiological degradation, reducing their ability to adapt to air pollution [60]. Compared to younger individuals, the elderly have lower immunity and antioxidant defense capabilities and more severe atherosclerosis, which increases their risk of acute effects from air pollution [61]. Due to increased blood viscosity from coronary atherosclerosis, the elderly are also a high-risk group for angina. Our results suggest that older adults should pay more attention to protective measures against air pollution. Wearing protective masks and staying indoors are two effective methods [62–64]. However, some studies have found opposite results, indicating that younger people are more affected by air pollution [65]. This discrepancy might be because younger individuals spend more time outdoors commuting and working. To understand the differential impact of air pollution on various age groups, further research is necessary. This will help develop better, more personalized prevention strategies.

Another important finding of this study is that the impact of air pollutants is higher during cold seasons compared to warm seasons. Some studies have shown that air pollution poses a greater risk during cooler seasons [35, 58]. Previous studies have reported that low temperatures can cause vasoconstriction, blood pressure fluctuations, and autonomic nervous system changes, which may contribute to angina onset [66]. Additionally, during cold seasons, coal combustion for heating leads to a significant increase in air pollution concentration, exacerbated by the unfavorable winter climate conditions in Lanzhou for the dispersion of air pollutants. However, some studies have indicated a stronger association between air pollution and angina during warm seasons [22, 50]. It is suggested that in warmer weather, individuals spend more time outdoors, increasing their exposure to outdoor air pollution, thus reducing misclassification of exposure derived from fixed monitoring stations. Moreover, increased blood circulation and sweating during warm seasons can lead to dehydration, elevated blood viscosity, and cholesterol levels, which may trigger angina events [67]. As a potential confounding factor and independent risk factor for angina, further research is needed to better understand the influence of air pollution on angina during different seasons.

Understanding the exposure-response relationship is crucial for formulating public health policies and setting air pollution limits. In this study, as air pollution concentrations increased, the relative risk of angina hospitalization showed an upward trend, with no apparent threshold. Even at relatively low concentrations, there could be an increased risk of angina hospitalization. These curves suggest that further mitigation of air pollution can significantly reduce the burden of angina hospitalizations. In 2021, the World Health Organization proposed stricter air quality standards than those in 2005 and recommended a series of actions to reduce air pollution, including the use of renewable energy, changing household heating methods, improving energy efficiency in urban planning and buildings, and promoting low-emission transportation methods [68]. Efforts should be intensified to implement the above measures to reduce the burden of future cardiovascular diseases.

Several limitations should be noted in our study. Firstly, the data on air pollution exposure was based on fixed-site monitoring stations, which may not accurately represent individual exposure levels. Secondly, confounding factors such as socioeconomic status and access to healthcare were not fully accounted for, which could influence the results. Thirdly, the exact proportion of the study sample to the total number of angina hospitalizations within the study area could not be determined, which may have impacted the comprehensiveness and representativeness of the results. Finally, this study did not account for indoor air pollution, outdated residential information, and population mobility, which could introduce bias into the results. In the future, more in-depth research can be conducted by expanding the scope of data collection, refining exposure assessment, and considering more confounding factors.

## Conclusion

Short-term exposure to PM_2.5_, SO_2_, NO_2_, and CO increases the risk of angina hospitalization, with evident lag effects. Women and the elderly are more susceptible to pollutants. The adverse effects of pollution are stronger in the cold season. Our study provides evidence about air pollution increasing hospitalization for angina. This research is crucial for preventing angina occurrences. Efforts should be made to improve urban environmental air pollution conditions and implement measures targeting different sensitive populations to reduce the risk of angina hospitalization.

### Electronic supplementary material

Below is the link to the electronic supplementary material.

Supplementary Material 1

## Data Availability

The data that support the findings of this study are available from Health Commission of Lanzhou City, a government agency but restrictions apply to the availability of these data, which were used under license for the current study, and so are not publicly available. Data are however available from the authors upon reasonable request and with permission of Health Commission of Lanzhou City, a government agency. After obtaining third-party permission, data analyzed during the current study are available from the corresponding author (ruany@lzu.edu.cn) on reasonable request. Meteorological data are available at http://data.cma.cn, which is managed by the National Meteorological Information Center of China, but researchers are required to register for free at the site to access the required data. Clinical admission data are not available to the public. R version 4.2.2 software was used in this study, and licenses and other information are available at https://www.R-project.org. The software package used to construct the distributed lag nonlinear model (DLNM) is publicly available at https://www.r-project.org/.
