# Supplementary material for: Effects of major air pollutants on angina hospitalizations: a correlation study
Source: BMC Public Health. 2024 Jul 15;24:1877. doi: 10.1186/s12889-024-19380-2 (PMC11247793; doi:10.1186/s12889-024-19380-2)
Supplement: Supplementary file 1 — Supplementary Material 1 [file 12889_2024_19380_MOESM1_ESM.docx]

**Effects of Major Air Pollutants on Angina Hospitalizations: A Correlation Study**

*Anning Zhu^1^, Yongqin Cao^2^, Chunlan Li^3^, Jingze Yu^1^, Miaoxin Liu^1^, Ke Xu^1^, Ye Ruan^1*^*

*^1^School of Public Health, Lanzhou University, Lanzhou730000, PR China*

*^2^Gansu Provincial Center for Disease Control and Prevention, 730000, PR China*

*^3^Third People's Hospital of Gansu Province, 730000, PR China*


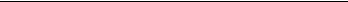


*Corresponding author: Ye Ruan Tel +86 931 8915191

E-mail address: ruany@lzu.edu.cn

**Contents of this file**

Table S1 to S7

Table S1 Spearman’s rank correlation coefficients between air pollutants and meteorological variables in Lanzhou, China, January 1, 2013 to December 31, 2020

| Variable | PM_2.5_ | PM_10_ | SO_2_ | NO_2_ | O_3_ | CO | Temp (℃) | RH (%) |
| --- | --- | --- | --- | --- | --- | --- | --- | --- |
| PM_2.5_ | 1 |  |  |  |  |  |  |  |
| PM_10_ | 0.850* | 1 |  |  |  |  |  |  |
| SO_2_ | 0.681* | 0.594* | 1 |  |  |  |  |  |
| NO_2_ | 0.372* | 0.363* | 0.430* | 1 |  |  |  |  |
| O_3_ | -0.341* | -0.156* | -0.407* | -0.005 | 1 |  |  |  |
| CO | 0.603* | 0.466* | 0.698* | 0.630* | -0.349* | 1 |  |  |
| Temp (℃) | -0.464* | -0.312* | -0.578* | -0.267* | 0.592* | -0.503* | 1 |  |
| RH (%) | -0.161* | -0.401* | -0.252* | -0.111* | -0.300* | 0.011 | -0.036 | 1 |

**P* < 0.05; Temp, temperature; RH, relative humidity

Table S2 Relative risk (95% CI) of angina hospitalization at different lag days associated with a 10 µg/m^3^ increase in air pollutant concentration (each 1 mg/m^3^ increase in CO) in a single pollutant model

| Lag days | PM_2.5_ | | PM_10_ | | SO_2_ | | NO_2_ | | O_3_ | | CO | |
| --- | --- | --- | --- | --- | --- | --- | --- | --- | --- | --- | --- | --- |
|  | RR | 95% CI | RR | 95% CI | RR | 95% CI | RR | 95% CI | RR | 95% CI | RR | 95% CI |
| Lag0 | 1.014 | (0.996,1.032) | 1.003 | (0.998,1.007) | 1.008 | (0.964,1.055) | 1.023 | (1.000,1.047)* | 1.001 | (0.987,1.015) | 1.047 | (0.968,1.133) |
| Lag1 | 0.988 | (0.968,1.008) | 0.997 | (0.991,1.002) | 1.033 | (0.982,1.087) | 1.008 | (0.981,1.036) | 1.001 | (0.987,1.016) | 1.001 | (0.913,1.099) |
| Lag2 | 1.012 | (0.993,1.032) | 1.001 | (0.996,1.006) | 1.001 | (0.952,1.053) | 0.997 | (0.970,1.025) | 0.988 | (0.973,1.003) | 1.050 | (0.956,1.153) |
| Lag3 | 1.019 | (1.000,1.038)* | 1.004 | (0.999,1.009) | 1.023 | (0.973,1.076) | 1.016 | (0.989,1.044) | 0.993 | (0.978,1.008) | 1.054 | (0.960,1.158) |
| Lag4 | 0.997 | (0.978,1.017) | 0.999 | (0.994,1.004) | 0.986 | (0.938,1.037) | 1.001 | (0.974,1.028) | 0.993 | (0.978,1.008) | 1.024 | (0.932,1.125) |
| Lag5 | 0.999 | (0.980,1.020) | 0.998 | (0.993,1.004) | 0.986 | (0.939,1.037) | 1.014 | (0.987,1.042) | 0.984 | (0.970,0.999)* | 0.967 | (0.881,1.063) |
| Lag6 | 1.003 | (0.983,1.024) | 1.001 | (0.995,1.006) | 1.027 | (0.977,1.079) | 1.017 | (0.989,1.045) | 0.999 | (0.984,1.014) | 1.082 | (0.985,1.189) |
| Lag7 | 1.009 | (0.991,1.028) | 1.004 | (0.999,1.008) | 0.989 | (0.946,1.033) | 0.983 | (0.960,1.007) | 1.007 | (0.993,1.021) | 0.925 | (0.853,1.003) |
| Lag0-1 | 1.002 | (0.983,1.021) | 0.999 | (0.994,1.005) | 1.041 | (0.989,1.096) | 1.031 | (1.004,1.058)* | 1.002 | (0.985,1.020) | 1.049 | (0.962,1.144) |
| Lag0-2 | 1.014 | (0.993,1.035) | 1.000 | (0.994,1.006) | 1.043 | (0.986,1.103) | 1.028 | (0.999,1.058) | 0.990 | (0.972,1.010) | 1.101 | (0.998,1.214) |
| Lag0-3 | 1.033 | (1.010,1.056)*  8 | 1.004 | (0.998,1.010) | 1.067 | (1.005,1.133)* | 1.044 | (1.012,1.077)* | 0.984 | (0.964,1.004) | 1.161 | (1.046,1.288)* |
| Lag0-4 | 1.030 | (1.006,1.055)* | 1.003 | (0.997,1.010) | 1.052 | (0.989,1.119) | 1.045 | (1.011,1.080)* | 0.977 | (0.956,0.998)* | 1.189 | (1.065,1.326)* |
| Lag0-5 | 1.030 | (1.004,1.056)* | 1.002 | (0.995,1.009) | 1.038 | (0.974,1.106) | 1.060 | (1.025,1.097)* | 0.962 | (0.941,0.983)* | 1.150 | (1.028,1.287)* |
| Lag0-6 | 1.033 | (1.006,1.060)* | 1.003 | (0.995,1.010) | 1.066 | (1.000,1.137)* | 1.078 | (1.041,1.117)* | 0.960 | (0.940,0.982)* | 1.244 | (1.109,1.397)* |
| Lag0-7 | 1.042 | (1.017,1.068)* | 1.006 | (0.999,1.013) | 1.054 | (0.996,1.116) | 1.060 | (1.026,1.094)* | 0.967 | (0.948,0.986)* | 1.151 | (1.037,1.277)* |

**P* < 0.05

Table S3 Relative risk (95% CI) of angina hospitalization stratified by sex at different lag days for each 10 µg/m^3^ increase in air pollutant concentration (each 1 mg/m^3^ increase in CO) in a single-pollutant model

| Sex | Lag days | PM_2.5_ | | PM_10_ | | SO_2_ | | NO_2_ | | | O_3_ | | | CO | | |
| --- | --- | --- | --- | --- | --- | --- | --- | --- | --- | --- | --- | --- | --- | --- | --- | --- |
|  |  | RR | 95% CI | RR | 95% CI | RR | 95% CI | RR | 95% CI | RR | | 95% CI | RR | | 95% CI |  |
| Man |  |  |  |  |  |  |  |  |  |  | |  |  | |  |  |
|  | Lag0 | 1.014 | (0.995,1.034) | 1.003 | (0.997,1.008) | 1.010 | (0.962,1.059) | 1.026 | (1.000,1.051)* | 1.001 | | (0.986,1.016) | 1.053 | | (0.968,1.146) |  |
|  | Lag1 | 0.982 | (0.961,1.004) | 0.996 | (0.990,1.002) | 1.026 | (0.972,1.083) | 1.002 | (0.973,1.032) | 1.000 | | (0.984,1.016) | 0.980 | | (0.887,1.082) |  |
|  | Lag2 | 1.016 | (0.995,1.037) | 1.002 | (0.996,1.007) | 1.004 | (0.951,1.059) | 1.002 | (0.973,1.032) | 0.989 | | (0.973,1.005) | 1.061 | | (0.959,1.173) |  |
|  | Lag3 | 1.016 | (0.996,1.036) | 1.004 | (0.998,1.009) | 1.011 | (0.959,1.067) | 1.009 | (0.980,1.039) | 0.995 | | (0.979,1.011) | 1.041 | | (0.942,1.151) |  |
|  | Lag4 | 0.998 | (0.978,1.019) | 1.000 | (0.994,1.005) | 0.982 | (0.931,1.036) | 0.999 | (0.970,1.028) | 0.994 | | (0.978,1.010) | 1.015 | | (0.918,1.122) |  |
|  | Lag5 | 1.001 | (0.980,1.023) | 0.999 | (0.993,1.005) | 1.000 | (0.949,1.054) | 1.018 | (0.989,1.048) | 0.984 | | (0.968,1.000) | 0.997 | | (0.902,1.102) |  |
|  | Lag6 | 1.005 | (0.984,1.027) | 1.001 | (0.995,1.007) | 1.029 | (0.976,1.084) | 1.014 | (0.985,1.044) | 0.997 | | (0.981,1.013) | 1.059 | | (0.957,1.171) |  |
|  | Lag7 | 1.007 | (0.988,1.027) | 1.003 | (0.999,1.008) | 0.996 | (0.950,1.044) | 0.988 | (0.963,1.013) | 1.008 | | (0.994,1.023) | 0.944 | | (0.866,1.030) |  |
|  | Lag0-1 | 0.997 | (0.976,1.017) | 0.998 | (0.993,1.004) | 1.036 | (0.981,1.094) | 1.028 | (0.999,1.057) | 1.001 | | (0.983,1.019) | 1.032 | | (0.940,1.132) |  |
|  | Lag0-2 | 1.012 | (0.990,1.035) | 1.000 | (0.994,1.006) | 1.040 | (0.979,1.104) | 1.030 | (0.998,1.062) | 0.990 | | (0.970,1.010) | 1.095 | | (0.986,1.216) |  |
|  | Lag0-3 | 1.029 | (1.005,1.053)* | 1.004 | (0.997,1.010) | 1.052 | (0.987,1.121) | 1.039 | (1.005,1.074)* | 0.985 | | (0.964,1.006) | 1.140 | | (1.019,1.274)* |  |
|  | Lag0-4 | 1.027 | (1.001,1.053)* | 1.003 | (0.996,1.010) | 1.033 | (0.967,1.103) | 1.038 | (1.002,1.075)* | 0.979 | | (0.957,1.001) | 1.156 | | (1.029,1.300)* |  |
|  | Lag0-5 | 1.028 | (1.001,1.056)* | 1.002 | (0.995,1.009) | 1.033 | (0.965,1.105) | 1.056 | (1.018,1.095)* | 0.963 | | (0.941,0.985)* | 1.153 | | (1.022,1.300)* |  |
|  | Lag0-6 | 1.033 | (1.005,1.062)* | 1.003 | (0.995,1.011) | 1.063 | (0.993,1.138) | 1.071 | (1.031,1.112)* | 0.960 | | (0.938,0.982)* | 1.221 | | (1.079,1.381)* |  |
|  | Lag0-7 | 1.040 | (1.014,1.068)* | 1.006 | (0.999,1.014) | 1.058 | (0.996,1.124) | 1.058 | (1.022,1.095)* | 0.968 | | (0.948,0.988)* | 1.152 | | (1.031,1.288)* |  |
| Woman |  |  |  |  |  |  |  |  |  |  | |  |  | |  |  |
|  | Lag0 | 1.013 | (0.994,1.033) | 1.003 | (0.998,1.008) | 1.006 | (0.959,1.055) | 1.019 | (0.994,1.044) | 1.002 | | (0.987,1.017) | 1.038 | | (0.956,1.128) |  |
|  | Lag1 | 0.997 | (0.976,1.018) | 0.998 | (0.992,1.004) | 1.044 | (0.990,1.102) | 1.017 | (0.988,1.046) | 1.004 | | (0.988,1.020) | 1.037 | | (0.940,1.143) |  |
|  | Lag2 | 1.006 | (0.985,1.027) | 0.999 | (0.994,1.005) | 0.998 | (0.946,1.052) | 0.989 | (0.962,1.018) | 0.987 | | (0.971,1.003) | 1.033 | | (0.936,1.140) |  |
|  | Lag3 | 1.023 | (1.003,1.043)* | 1.005 | (1.000,1.010)* | 1.042 | (0.988,1.100) | 1.027 | (0.998,1.056) | 0.990 | | (0.974,1.006) | 1.074 | | (0.973,1.185) |  |
|  | Lag4 | 0.996 | (0.976,1.017) | 0.999 | (0.993,1.004) | 0.993 | (0.942,1.048) | 1.004 | (0.975,1.033) | 0.992 | | (0.976,1.008) | 1.040 | | (0.942,1.149) |  |
|  | Lag5 | 0.997 | (0.976,1.019) | 0.998 | (0.992,1.004) | 0.964 | (0.915,1.017) | 1.009 | (0.981,1.038) | 0.985 | | (0.969,1.001) | 0.922 | | (0.835,1.017) |  |
|  | Lag6 | 1.000 | (0.979,1.022) | 1.000 | (0.994,1.006) | 1.024 | (0.972,1.079) | 1.021 | (0.992,1.051) | 1.002 | | (0.986,1.018) | 1.121 | | (1.015,1.238)* |  |
|  | Lag7 | 1.013 | (0.994,1.032) | 1.004 | (0.999,1.009) | 0.977 | (0.932,1.024) | 0.975 | (0.952,1.000) | 1.005 | | (0.990,1.019) | 0.894 | | (0.821,0.974) |  |
|  | Lag0-1 | 1.010 | (0.990,1.031) | 1.001 | (0.995,1.006) | 1.050 | (0.995,1.109) | 1.036 | (1.008,1.064)* | 1.005 | | (0.987,1.024) | 1.077 | | (0.983,1.179) |  |
|  | Lag0-2 | 1.016 | (0.994,1.039) | 1.000 | (0.994,1.006) | 1.048 | (0.987,1.112) | 1.025 | (0.994,1.056) | 0.992 | | (0.972,1.013) | 1.112 | | (1.004,1.232)* |  |
|  | Lag0-3 | 1.039 | (1.016,1.064)* | 1.005 | (0.999,1.012) | 1.092 | (1.026,1.163)* | 1.052 | (1.019,1.087)* | 0.982 | | (0.960,1.004) | 1.194 | | (1.070,1.333)* |  |
|  | Lag0-4 | 1.036 | (1.010,1.062)* | 1.004 | (0.997,1.011) | 1.085 | (1.017,1.158)* | 1.056 | (1.021,1.093)* | 0.974 | | (0.952,0.997)* | 1.242 | | (1.107,1.393)* |  |
|  | Lag0-5 | 1.032 | (1.005,1.060)* | 1.002 | (0.994,1.009) | 1.046 | (0.979,1.119) | 1.066 | (1.029,1.105)* | 0.960 | | (0.937,0.982)* | 1.145 | | (1.017,1.288)* |  |
|  | Lag0-6 | 1.033 | (1.004,1.062)* | 1.002 | (0.994,1.009) | 1.072 | (1.001,1.147)* | 1.089 | (1.049,1.130)* | 0.961 | | (0.939,0.984)* | 1.284 | | (1.136,1.450)* |  |
|  | Lag0-7 | 1.046 | (1.019,1.073)* | 1.006 | (0.998,1.013) | 1.047 | (0.986,1.112) | 1.062 | (1.026,1.099)* | 0.966 | | (0.945,0.986)* | 1.148 | | (1.028,1.281)* |  |

**P* < 0.05

Table S4 Relative risk (95% CI) of angina hospitalization stratified by age at different lag days for each 10 µg/m^3^ increase in air pollutant concentration (each 1 mg/m^3^ increase in CO) in a single-pollutant model

| Age | Lag days | PM_2.5_ | | PM_10_ | | SO_2_ | | NO_2_ | | O_3_ | | CO | |
| --- | --- | --- | --- | --- | --- | --- | --- | --- | --- | --- | --- | --- | --- |
|  |  | RR | 95% CI | RR | 95% CI | RR | 95% CI | RR | 95% CI | RR | 95% CI | RR | 95% CI |
| < 65y |  |  |  |  |  |  |  |  |  |  |  |  |  |
|  | Lag0 | 1.011 | (0.991,1.031) | 1.002 | (0.997,1.008) | 0.994 | (0.946,1.044) | 1.014 | (0.989,1.041) | 1.007 | (0.991,1.022) | 1.026 | (0.941,1.119) |
|  | Lag1 | 0.984 | (0.961,1.006) | 0.995 | (0.989,1.002) | 1.039 | (0.983,1.099) | 1.003 | (0.973,1.034) | 1.000 | (0.983,1.016) | 0.987 | (0.892,1.093) |
|  | Lag2 | 1.018 | (0.997,1.039) | 1.002 | (0.996,1.007) | 1.016 | (0.961,1.074) | 1.005 | (0.975,1.035) | 0.990 | (0.974,1.007) | 1.093 | (0.985,1.211) |
|  | Lag3 | 1.018 | (0.997,1.038) | 1.004 | (0.999,1.009) | 1.010 | (0.955,1.067) | 1.014 | (0.984,1.045) | 0.988 | (0.971,1.004) | 1.035 | (0.933,1.147) |
|  | Lag4 | 0.996 | (0.975,1.018) | 0.999 | (0.993,1.005) | 0.988 | (0.935,1.044) | 1.006 | (0.976,1.037) | 0.987 | (0.971,1.003) | 1.021 | (0.920,1.132) |
|  | Lag5 | 1.000 | (0.979,1.023) | 0.998 | (0.992,1.004) | 0.982 | (0.930,1.036) | 1.012 | (0.982,1.042) | 0.987 | (0.970,1.003) | 0.972 | (0.877,1.078) |
|  | Lag6 | 0.998 | (0.976,1.021) | 1.001 | (0.995,1.007) | 1.030 | (0.975,1.087) | 1.016 | (0.986,1.048) | 0.999 | (0.983,1.015) | 1.070 | (0.965,1.186) |
|  | Lag7 | 1.011 | (0.991,1.031) | 1.004 | (0.999,1.009) | 0.989 | (0.942,1.038) | 0.985 | (0.959,1.011) | 1.008 | (0.993,1.023) | 0.928 | (0.849,1.015) |
|  | Lag0-1 | 0.994 | (0.973,1.016) | 0.998 | (0.992,1.004) | 1.033 | (0.976,1.092) | 1.018 | (0.989,1.047) | 1.006 | (0.988,1.025) | 1.013 | (0.921,1.114) |
|  | Lag0-2 | 1.012 | (0.989,1.035) | 0.999 | (0.993,1.006) | 1.049 | (0.986,1.116) | 1.022 | (0.990,1.056) | 0.996 | (0.976,1.017) | 1.106 | (0.994,1.232) |
|  | Lag0-3 | 1.030 | (1.005,1.055)* | 1.003 | (0.997,1.010) | 1.059 | (0.992,1.131) | 1.036 | (1.001,1.072)* | 0.984 | (0.962,1.006) | 1.145 | (1.021,1.284)* |
|  | Lag0-4 | 1.026 | (0.999,1.053) | 1.002 | (0.995,1.010) | 1.046 | (0.978,1.119) | 1.043 | (1.006,1.081)* | 0.971 | (0.949,0.994)* | 1.168 | (1.037,1.317)* |
|  | Lag0-5 | 1.026 | (0.998,1.055) | 1.000 | (0.993,1.008) | 1.027 | (0.958,1.101) | 1.055 | (1.016,1.095)* | 0.958 | (0.935,0.981)* | 1.136 | (1.004,1.285)* |
|  | Lag0-6 | 1.024 | (0.995,1.054) | 1.001 | (0.993,1.009) | 1.058 | (0.986,1.134) | 1.072 | (1.031,1.115)* | 0.957 | (0.934,0.980)* | 1.215 | (1.070,1.380)* |
|  | Lag0-7 | 1.035 | (1.007,1.064)* | 1.005 | (0.997,1.013) | 1.046 | (0.983,1.113) | 1.056 | (1.019,1.094)* | 0.965 | (0.944,0.985)* | 1.128 | (1.006,1.265)* |
| ≥ 65y |  |  |  |  |  |  |  |  |  |  |  |  |  |
|  | Lag0 | 1.017 | (0.999,1.035) | 1.003 | (0.998,1.008) | 1.023 | (0.977,1.071) | 1.032 | (1.007,1.056)* | 0.995 | (0.981,1.010) | 1.070 | (0.986,1.160) |
|  | Lag1 | 0.992 | (0.972,1.013) | 0.998 | (0.992,1.003) | 1.026 | (0.974,1.081) | 1.012 | (0.984,1.041) | 1.003 | (0.988,1.019) | 1.016 | (0.923,1.117) |
|  | Lag2 | 1.007 | (0.987,1.027) | 1.000 | (0.995,1.005) | 0.987 | (0.937,1.039) | 0.990 | (0.963,1.018) | 0.986 | (0.971,1.001) | 1.009 | (0.917,1.111) |
|  | Lag3 | 1.020 | (1.001,1.040)* | 1.004 | (0.999,1.009) | 1.037 | (0.985,1.092) | 1.018 | (0.990,1.047) | 0.999 | (0.983,1.014) | 1.073 | (0.975,1.181) |
|  | Lag4 | 0.999 | (0.979,1.018) | 0.999 | (0.994,1.005) | 0.985 | (0.935,1.037) | 0.995 | (0.968,1.023) | 0.999 | (0.984,1.015) | 1.028 | (0.933,1.132) |
|  | Lag5 | 0.999 | (0.978,1.019) | 0.999 | (0.993,1.004) | 0.992 | (0.942,1.043) | 1.017 | (0.989,1.046) | 0.982 | (0.967,0.998)* | 0.962 | (0.874,1.059) |
|  | Lag6 | 1.008 | (0.988,1.029) | 1.001 | (0.995,1.006) | 1.024 | (0.973,1.077) | 1.017 | (0.989,1.046) | 0.999 | (0.984,1.014) | 1.094 | (0.993,1.205) |
|  | Lag7 | 1.008 | (0.990,1.027) | 1.003 | (0.999,1.008) | 0.989 | (0.944,1.035) | 0.982 | (0.959,1.006) | 1.006 | (0.992,1.020) | 0.921 | (0.847,1.001) |
|  | Lag0-1 | 1.009 | (0.989,1.029) | 1.001 | (0.995,1.006) | 1.050 | (0.996,1.107) | 1.044 | (1.017,1.072)* | 0.999 | (0.981,1.017) | 1.086 | (0.994,1.187) |
|  | Lag0-2 | 1.016 | (0.994,1.038) | 1.000 | (0.995,1.006) | 1.036 | (0.978,1.098) | 1.034 | (1.003,1.064)* | 0.985 | (0.965,1.004) | 1.096 | (0.992,1.212) |
|  | Lag0-3 | 1.036 | (1.013,1.060)* | 1.005 | (0.999,1.011) | 1.074 | (1.010,1.143)* | 1.052 | (1.019,1.086)* | 0.983 | (0.962,1.004) | 1.177 | (1.057,1.310)* |
|  | Lag0-4 | 1.035 | (1.010,1.060)* | 1.004 | (0.998,1.011) | 1.058 | (0.993,1.127) | 1.047 | (1.013,1.083)* | 0.982 | (0.961,1.004) | 1.209 | (1.081,1.353)* |
|  | Lag0-5 | 1.033 | (1.007,1.060)* | 1.003 | (0.996,1.010) | 1.049 | (0.983,1.119) | 1.065 | (1.029,1.103)* | 0.965 | (0.944,0.987)* | 1.164 | (1.037,1.306)* |
|  | Lag0-6 | 1.042 | (1.014,1.070)* | 1.004 | (0.997,1.011) | 1.074 | (1.005,1.147)* | 1.083 | (1.045,1.123)* | 0.964 | (0.943,0.986)* | 1.273 | (1.131,1.433)* |
|  | Lag0-7 | 1.050 | (1.024,1.077)* | 1.007 | (1.000,1.014)* | 1.061 | (1.001,1.125)* | 1.064 | (1.029,1.099)* | 0.969 | (0.950,0.989)* | 1.173 | (1.054,1.305)* |

**P* < 0.05

Table S5 Relative risk (95% CI) of angina hospitalization stratified by season at different lag days for each 10 µg/m^3^ increase in air pollutant concentration (each 1 mg/m^3^ increase in CO) in a single-pollutant model

| Season | Lag days | PM_2.5_ | | PM_10_ | | SO_2_ | | NO_2_ | | O_3_ | | CO | |
| --- | --- | --- | --- | --- | --- | --- | --- | --- | --- | --- | --- | --- | --- |
|  |  | RR | 95% CI | RR | 95% CI | RR | 95% CI | RR | 95% CI | RR | 95% CI | RR | 95% CI |
| Cold |  |  |  |  |  |  |  |  |  |  |  |  |  |
|  | Lag0 | 1.005 | (0.980,1.031) | 1.001 | (0.994,1.008) | 1.032 | (0.977,1.089) | 1.047 | (1.017,1.077)* | 1.009 | (0.984,1.034) | 1.078 | (0.988,1.177) |
|  | Lag1 | 0.994 | (0.964,1.024) | 0.999 | (0.991,1.007) | 1.024 | (0.962,1.090) | 0.997 | (0.964,1.032) | 0.996 | (0.969,1.023) | 0.962 | (0.867,1.068) |
|  | Lag2 | 0.998 | (0.969,1.028) | 0.998 | (0.990,1.006) | 1.028 | (0.965,1.094) | 1.010 | (0.976,1.045) | 0.967 | (0.941,0.994)* | 1.087 | (0.977,1.208) |
|  | Lag3 | 1.028 | (0.998,1.058) | 1.003 | (0.995,1.011) | 1.033 | (0.970,1.100) | 1.018 | (0.983,1.054) | 0.990 | (0.964,1.017) | 1.064 | (0.956,1.185) |
|  | Lag4 | 0.994 | (0.966,1.024) | 0.999 | (0.991,1.006) | 0.998 | (0.939,1.062) | 1.003 | (0.969,1.038) | 0.979 | (0.952,1.006) | 1.063 | (0.954,1.184) |
|  | Lag5 | 1.003 | (0.973,1.033) | 0.997 | (0.989,1.006) | 1.003 | (0.944,1.066) | 1.011 | (0.977,1.046) | 0.974 | (0.948,1.002) | 0.979 | (0.880,1.088) |
|  | Lag6 | 1.006 | (0.977,1.037) | 1.003 | (0.995,1.011) | 1.028 | (0.967,1.093) | 1.026 | (0.991,1.062) | 0.971 | (0.945,0.999)* | 1.109 | (0.997,1.234) |
|  | Lag7 | 0.987 | (0.962,1.012) | 0.998 | (0.991,1.005) | 0.981 | (0.930,1.035) | 0.973 | (0.944,1.002) | 1.006 | (0.982,1.031) | 0.926 | (0.845,1.013) |
|  | Lag0-1 | 0.999 | (0.972,1.026) | 1.000 | (0.993,1.008) | 1.056 | (0.994,1.122) | 1.044 | (1.011,1.078)* | 1.005 | (0.976,1.035) | 1.038 | (0.942,1.143) |
|  | Lag0-2 | 0.997 | (0.969,1.027) | 0.998 | (0.990,1.006) | 1.085 | (1.015,1.161)* | 1.054 | (1.017,1.093)* | 0.972 | (0.940,1.005) | 1.127 | (1.009,1.260)* |
|  | Lag0-3 | 1.025 | (0.994,1.057) | 1.001 | (0.992,1.009) | 1.121 | (1.044,1.204)* | 1.073 | (1.033,1.115)* | 0.962 | (0.928,0.998)* | 1.200 | (1.067,1.349)* |
|  | Lag0-4 | 1.019 | (0.986,1.053) | 0.999 | (0.990,1.008) | 1.119 | (1.040,1.205)* | 1.077 | (1.035,1.120)* | 0.942 | (0.907,0.978)* | 1.275 | (1.127,1.442)* |
|  | Lag0-5 | 1.022 | (0.987,1.058) | 0.997 | (0.987,1.006) | 1.123 | (1.041,1.212)* | 1.089 | (1.044,1.135)* | 0.918 | (0.883,0.953)* | 1.248 | (1.099,1.417)* |
|  | Lag0-6 | 1.029 | (0.992,1.066) | 1.000 | (0.990,1.010) | 1.155 | (1.068,1.249)* | 1.117 | (1.069,1.167)* | 0.891 | (0.858,0.926)* | 1.384 | (1.212,1.580)* |
|  | Lag0-7 | 1.015 | (0.982,1.049) | 0.998 | (0.989,1.007) | 1.133 | (1.056,1.215)* | 1.086 | (1.043,1.132)* | 0.897 | (0.867,0.928)* | 1.281 | (1.135,1.445)* |
| Warm |  |  |  |  |  |  |  |  |  |  |  |  |  |
|  | Lag0 | 1.015 | (0.991,1.040) | 1.003 | (0.997,1.010) | 0.965 | (0.880,1.059) | 0.973 | (0.932,1.015) | 1.001 | (0.984,1.018) | 1.016 | (0.814,1.268) |
|  | Lag1 | 0.980 | (0.953,1.008) | 0.994 | (0.987,1.002) | 1.042 | (0.950,1.144) | 1.016 | (0.969,1.065) | 1.000 | (0.982,1.018) | 1.231 | (0.977,1.550) |
|  | Lag2 | 1.015 | (0.989,1.043) | 1.003 | (0.996,1.010) | 0.917 | (0.835,1.008) | 0.947 | (0.904,0.992) | 0.995 | (0.978,1.014) | 0.786 | (0.620,0.995) |
|  | Lag3 | 1.011 | (0.986,1.037) | 1.004 | (0.998,1.011) | 1.005 | (0.916,1.103) | 1.003 | (0.957,1.050) | 0.999 | (0.981,1.017) | 0.910 | (0.717,1.154) |
|  | Lag4 | 0.990 | (0.964,1.016) | 0.998 | (0.991,1.004) | 0.942 | (0.859,1.033) | 0.998 | (0.953,1.045) | 1.008 | (0.990,1.027) | 0.845 | (0.669,1.069) |
|  | Lag5 | 0.992 | (0.965,1.020) | 0.998 | (0.991,1.006) | 0.991 | (0.904,1.086) | 1.030 | (0.983,1.079) | 0.985 | (0.967,1.003) | 0.991 | (0.786,1.250) |
|  | Lag6 | 0.995 | (0.966,1.025) | 0.996 | (0.988,1.004) | 1.010 | (0.923,1.105) | 0.991 | (0.946,1.038) | 1.005 | (0.988,1.023) | 1.056 | (0.836,1.333) |
|  | Lag7 | 1.022 | (0.997,1.048) | 1.006 | (1.000,1.013)* | 1.057 | (0.971,1.151) | 0.986 | (0.947,1.027) | 1.001 | (0.985,1.017) | 1.034 | (0.833,1.284) |
|  | Lag0-1 | 0.995 | (0.964,1.026) | 0.998 | (0.990,1.006) | 1.006 | (0.894,1.132) | 0.988 | (0.939,1.040) | 1.001 | (0.980,1.022) | 1.251 | (0.950,1.648) |
|  | Lag0-2 | 1.010 | (0.976,1.045) | 1.001 | (0.992,1.009) | 0.923 | (0.808,1.055) | 0.936 | (0.884,0.991) | 0.996 | (0.973,1.020) | 0.983 | (0.719,1.343) |
|  | Lag0-3 | 1.021 | (0.984,1.060) | 1.005 | (0.996,1.014) | 0.928 | (0.804,1.070) | 0.938 | (0.881,1.000) | 0.995 | (0.970,1.021) | 0.894 | (0.640,1.248) |
|  | Lag0-4 | 1.011 | (0.971,1.052) | 1.002 | (0.992,1.013) | 0.874 | (0.752,1.016) | 0.937 | (0.877,1.001) | 1.003 | (0.977,1.031) | 0.755 | (0.535,1.067) |
|  | Lag0-5 | 1.003 | (0.960,1.048) | 1.001 | (0.990,1.012) | 0.866 | (0.741,1.012) | 0.965 | (0.900,1.034) | 0.988 | (0.961,1.017) | 0.749 | (0.528,1.062) |
|  | Lag0-6 | 0.998 | (0.953,1.045) | 0.997 | (0.985,1.009) | 0.874 | (0.747,1.023) | 0.956 | (0.889,1.028) | 0.994 | (0.965,1.023) | 0.790 | (0.558,1.118) |
|  | Lag0-7 | 1.020 | (0.974,1.068) | 1.003 | (0.991,1.015) | 0.924 | (0.796,1.074) | 0.943 | (0.880,1.010) | 0.994 | (0.967,1.022) | 0.817 | (0.595,1.122) |

**P* < 0.05

Table S6 Adjusted relative risk (95% CI) of angina hospitalization for exposure to PM_2.5_ (lag0-7), PM_10_ (lag0-7), SO_2_ (lag0-3), NO_2_ (lag0-6), O_3_ (lag0-6), and CO (lag0-6) in two-pollutant model

| Air pollutant | Model | RR | 95% CI |
| --- | --- | --- | --- |
| PM_2.5_ (lag0-7) | PM_2.5_ | 1.043 | (1.017,1.068)* |
|  | PM_2.5_ + SO_2_ | 1.041 | (1.004,1.069)* |
|  | PM_2.5_ + NO_2_ | 1.031 | (1.004,1.058)* |
|  | PM_2.5_ + O_3_ | 1.039 | (1.013,1.066)* |
|  | PM_2.5_ +CO | 1.035 | (1.006,1.065)* |
| PM_10_ (lag0-7) | PM_10_ | 1.006 | (0.999,1.013) |
|  | PM_10_ + SO_2_ | 1.005 | (0.997,1.013) |
|  | PM_10_ + NO_2_ | 1.004 | (0.997,1.011) |
|  | PM_10_ + O_3_ | 1.005 | (0.998,1.012) |
|  | PM_10_ +CO | 1.005 | (0.998,1.012) |
| SO_2_ (lag0-3) | SO_2_ | 1.067 | (1.005,1.133)* |
|  | SO_2_ + PM_2.5_ | 1.054 | (0.990,1.122) |
|  | SO_2_ + PM_10_ | 1.065 | (1.002,1.131)* |
|  | SO_2_ + NO_2_ | 1.026 | (1.002,1.096)* |
|  | SO_2_ + O_3_ | 1.072 | (1.009,1.138)* |
|  | SO_2_ + CO | 1.025 | (0.951,1.106) |
| NO_2_ (lag0-6) | NO_2_ | 1.078 | (1.041,1.117)* |
|  | NO_2_ + PM_2.5_ | 1.074 | (1.036,1.114)* |
|  | NO_2_ + PM_10_ | 1.078 | (1.040,1.117)* |
|  | NO_2_ + SO_2_ | 1.082 | (1.040,1.124)* |
|  | NO_2_ + O_3_ | 1.071 | (1.034,1.110)* |
|  | NO_2_ + CO | 1.076 | (1.034,1.120)* |
| O_3_ (lag0-6) | O_3_ | 0.961 | (0.940,0.982) |
|  | O_3_ + PM_2.5_ | 0.962 | (0.942,0.983) |
|  | O_3_ + PM_10_ | 0.962 | (0.942,0.983) |
|  | O_3_ + SO_2_ | 0.991 | (0.981,1.000) |
|  | O_3_ + NO_2_ | 0.964 | (0.942,0.985) |
|  | O_3_ + CO | 0.963 | (0.943,0.985) |
| CO (lag0-6) | CO | 1.244 | (1.109,1.397)* |
|  | CO + PM_2.5_ | 1.214 | (1.062,1.388)* |
|  | CO + PM_10_ | 1.245 | (1.108,1.399)* |
|  | CO + SO_2_ | 1.337 | (1.120,1.596)* |
|  | CO + NO_2_ | 1.096 | (1.108,1.282)* |
|  | CO + O_3_ | 1.206 | (1.071,1.359)* |

**P* < 0.05

We selected the lag days that have significant and representative effects on angina hospitalizations. The chosen lag days are as follows: lag0-7 for PM_2.5_, lag0-7 for PM_10_, lag0-3 for SO_2_, lag0-6 for NO_2_, lag0-6 for O_3_, and lag0-6 for CO. These lag days represent the main lag effects of the pollutants on angina hospitalizations.

Table S7 Relative risk of angina admission after adjusting for different degree of freedom (6-10)

| Degree of freedom | PM_2.5_ (lag0-7) | | PM_10_ (lag0-7) | | SO_2_ (lag0-3) | | NO_2_ (lag0-6) | | O_3_ (lag0-6) | | CO (lag0-6) | |
| --- | --- | --- | --- | --- | --- | --- | --- | --- | --- | --- | --- | --- |
|  | RR | 95% CI | RR | 95% CI | RR | 95% CI | RR | 95% CI | RR | 95% CI | RR | 95% CI |
| *df* = 6 | 1.041 | (1.016,1.067)* | 1.006 | (0.999,1.013) | 1.069 | (1.007,1.135)* | 1.082 | (1.045,1.120)* | 0.963 | (0.942,0.984)* | 1.244 | (1.108,1.396)* |
| *df* = 7 | 1.042 | (1.017,1.068)* | 1.006 | (0.999,1.013) | 1.067 | (1.005,1.133)* | 1.078 | (1.041,1.117)* | 0.960 | (0.940,0.982)* | 1.244 | (1.109,1.397)* |
| *df* = 8 | 1.048 | (1.022,1.074)* | 1.007 | (1.000,1.014)* | 1.067 | (1.005,1.133)* | 1.077 | (1.040,1.116)* | 0.961 | (0.940,0.982)* | 1.250 | (1.114,1.403)* |
| *df* = 9 | 1.044 | (1.019,1.070)* | 1.006 | (0.999,1.013) | 1.065 | (1.003,1.131)* | 1.077 | (1.039,1.115)* | 0.962 | (0.941,0.983)* | 1.242 | (1.107,1.394)* |
| *df* = 10 | 1.050 | (1.024,1.076)* | 1.007 | (1.000,1.014) | 1.065 | (1.003,1.131)* | 1.076 | (1.039,1.114)* | 0.962 | (0.941,0.984)* | 1.249 | (1.113,1.402)* |

**P* < 0.05

We selected the lag days that have significant and representative effects on angina hospitalizations. The chosen lag days are as follows: lag0-7 for PM_2.5_, lag0-7 for PM_10_, lag0-3 for SO_2_, lag0-6 for NO_2_, lag0-6 for O_3_, and lag0-6 for CO. These lag days represent the main lag effects of the pollutants on angina hospitalizations.
